# Comparison of GeneXpert MRSA/SA ETA assay with semi-quantitative and quantitative cultures and *nuc* gene-based qPCR for detection of *Staphylococcus aureus* in endotracheal aspirate samples

**DOI:** 10.1186/s13756-018-0460-8

**Published:** 2019-01-05

**Authors:** Jasmine Coppens, Liesbet Van Heirstraeten, Alexey Ruzin, Li Yu, Leen Timbermont, Christine Lammens, Veerle Matheeussen, Michael McCarthy, Philippe Jorens, Margareta Ieven, Samir Kumar-Singh, Herman Goossens, Surbhi Malhotra-Kumar

**Affiliations:** 1 Laboratory of Medical Microbiology, Vaccine & Infectious Disease Institute, University of Antwerp, Universiteitsplein 1, D.S.6.23, 2610 Wilrijk, Belgium; 2 MedImmune, Gaithersburg, USA; 3 Laboratory of Clinical Microbiology, Antwerp University Hospital, Edegem, Belgium; 4 Department of Critical Care Medicine, Antwerp University Hospital, Edegem, Belgium; 5 Molecular Pathology Group, Cell Biology & Histology, University of Antwerp, Wilrijk, Belgium

**Keywords:** Cepheid, Real-time PCR, Rapid diagnostics, VAP, Ventilator-associated pneumonia, COLOREX™ staph aureus, Chromogenic medium

## Abstract

**Introduction:**

*Staphylococcus aureus (S. aureus)* is a common cause of ventilator-associated pneumonia. Rapid and accurate detection of lower respiratory tract colonization and/or infection with *S. aureus* may inform targeted preventive and therapeutic strategies. To investigate this, we compared semi-quantitative (SQ)-culture results from 79 endotracheal aspirates (ETA) collected from mechanically-ventilated patients, to two culture and two non-culture-based methods for detection of *S. aureus*.

**Methods:**

ETA analyzed by routine SQ-culture on blood and colistin-nalidixic-acid agar was compared to: (i) quantitative (Q-) culture on chromogenic COLOREX™ Staph aureus; (ii) enrichment in brain-heart-infusion broth followed by plating on blood agar and COLOREX™; (iii) *nuc*-based TaqMan qPCR, and (iv) GeneXpert MRSA/SA ETA assay.

**Results:**

Of the 79 ETA samples analyzed by SQ-culture, 39 samples were positive, and 40 negative for *S. aureus*. Two samples negative for *S. aureus* by SQ-culture were, however, *S. aureus*-positive by the other four methods and were considered positive. Appending these two samples as positive in the SQ-culture results, sensitivities−specificities for Q-culture, enrichment-culture, TaqMan qPCR and GeneXpert were 100–95, 100–92, 100–53% and 100% − 100, respectively. The lower specificities of Q-culture, enrichment-culture, and TaqMan qPCR was because of their higher sensitivities, although TaqMan qPCR also detected *S. aureus*-specific extracellular DNA.

**Conclusion:**

This first evaluation of the GeneXpert MRSA/SA ETA assay with ETA samples found it to be highly sensitive, specific, user-friendly (hands-on time ~ 5 min.), and rapid (~ 66 min. assay time). Where this equipment is not available, we recommend implementing more sensitive culture-based methods for improved *S. aureus* detection in ETA samples.

**Electronic supplementary material:**

The online version of this article (10.1186/s13756-018-0460-8) contains supplementary material, which is available to authorized users.

## Introduction

Methicillin-sensitive and -resistant *Staphylococcus aureus* (MSSA and MRSA) cause life-threatening infections in both high-risk and in healthy individuals [1]. One of the most common nosocomial infections where *S. aureus* is an important causative agent is ventilator-associated pneumonia (VAP) [2]. VAP not only results in a substantial increase in morbidity and mortality but also in a costly prolongation of patient bed days. The classical definition of VAP is based on clinical signs, a new infiltrate on the chest X-ray and, importantly, the detection of the causative pathogen from respiratory samples [3, 4].

Prior colonization with potential pathogens plays a significant role in the development of nosocomial infections [5]. For instance, intensive care unit (ICU) patients with *S. aureus* colonization have up to a 15-fold higher risk of developing VAP compared to non-colonized patients [6]. These data suggest that pathogen identification by surveillance culture could represent a preemptive strategy for VAP. ETAs have the advantage of being a noninvasive sample that can be obtained rapidly and repeatedly with fewer complications and resources, and are therefore commonly used for routine microbiological surveillance cultures [7].

While culture of pathogen remains the gold standard, molecular tests that typically have a shorter turnaround time can drastically decrease the critical time-to-initiation of preventive and therapeutic strategies, including the initiation of the appropriate antibacterial treatment. A recently introduced molecular test, currently utilized for research purposes only, that directly detects *S. aureus* from ETA samples is the GeneXpert SA/MRSA ETA assay. Here, we evaluated and compared the GeneXpert assay to culture and non-culture based methods for detection of *S. aureus* in ETA samples.

## Materials and methods

### Study design and sample collection

The study was designed to assess the performance of a rapid screening test, the GeneXpert MRSA/SA ETA assay, in patients at risk of developing VAP. During May 2017–February 2018, ETA samples were collected from mechanically ventilated adult patients at the ICU of the University Hospital of Antwerp, either as surveillance cultures or as routine samples obtained in patients with suspicion of (pulmonary) infection. ETAs were analysed immediately at the Clinical Microbiology laboratory of the University Hospital of Antwerp using quadrant-based, semi-quantitative (SQ)-culture on blood agar, chocolate agar, McConkey and colistin-nalidixic acid agar (CNA, Oxoid) and bacterial growth was evaluated after 24 h of incubation [8]. Based on quadrant growth, SQ-culture method categorizes positive samples as light (growth in quadrant 1), moderate (growth in quadrants 2 and also 3) and heavy (growth in all four quadrants). For every *S. aureus*-positive sample studied, a negative sample was also included. At the end of study period, 79 samples were collected as *S. aureus* negative (*n* = 40), light (*n* = 19), moderate (*n* = 10) and heavy (*n* = 10) (Additional file 1: Table S1). SQ-culture was followed by disk diffusion with cefoxitin (4 μg/ml). The samples were further processed within 48 h after ETA collection with different methods for *S. aureus* detection and quantitation at the Laboratory of Medical Microbiology, University of Antwerp. Methods used were: GeneXpert MRSA/SA ETA Assay; quantitative (Q-) culture on chromogenic COLOREX™ Staph aureus; enrichment broth followed by blood agar and COLOREX plating; and TaqMan qPCR targeting the single-copy thermostable nuclease (*nuc*) gene on extracted DNA. In case needed, the samples were stored at 4 °C until further processing (maximum 48 h). The study was approved by the ethics committee of the Antwerp University Hospital (Belgian registration number B300201629199).

### GeneXpert assay

The GeneXpert assay is currently available for research (RMRSA/SA-ETA-10, Cepheid, USA) and was performed according to manufacturer’s instructions. Briefly, ETA samples were adsorbed onto a Cepheid collection device swab (COPAN- 900-0370, USA), dissolved in elution buffer and vortexed at high speed for 10 s (Scientific Industries Inc., USA). The entire content of the elution reagent was transferred to the cartridge and analysed by GeneXpert® Dx system v4.7b (Cepheid, USA). The overall process of extraction, amplification, and detection of the targets was completed in 66 min. The primers and probes in the MRSA/SA ETA assay detect sequences within the staphylococcal protein A (*spa*) gene, the methicillin resistance gene (*mecA*), and the staphylococcal cassette chromosome (*SCCmec*) inserted into the *S. aureus* chromosomal *attB* insertion site [9]. Samples were reported by GeneXpert as either *S. aureus* “detected” (Ct 3–36) or “not detected”.

### Sample preparation for comparator culture-based and qPCR methods

After initiating the GeneXpert assay, the remaining ETA sample was homogenised by blending and liquefaction with N-acetylcysteine. All samples were blended with the dispersing instrument T10 basic ULTRA-TURRAX (IKA, Staufen, Germany) for 10 s at maximum speed on ice. Depending on the viscosity of the sample, checked by visual inspection, the blending time was increased by steps of 20 s, with a maximum blending time of 60 s. After blending, the samples were liquefied with lysomucil (10% N-acetylcysteine, Zambon, Milan, Italy). 3 ml lysomucil was dissolved in 12 ml phosphate buffered saline (PBS), and an equal amount in volume (~ 300 μL) of liquefying reagent was added as 1:1 to the sample, and vortexed at full speed for 10 s. After incubation at 37 °C for 15 min, the samples were vortexed again at full speed for 10 s, and the incubation step was repeated.

### Quantitative cultures

Serial dilutions of the liquefied samples were spiral-plated (Eddy Jet, program 6; 50 μL logarithmic spreading; IUL, Spain) on the chromogenic COLOREX™ Staph aureus medium (bioTRADING, The Netherlands) and on blood agar (Columbia II Agar Base, Oxoid, UK with 5% defibrinated horse blood). After 24 h of incubation, pink to mauve *S. aureus* colonies on the COLOREX*™* Staph aureus were counted and *S. aureus* loads were calculated as colony forming units (CFU)/ml for each sample. At least one pink to mauve colony per plate was speciated by MALDI-TOF (Bruker, USA) and stored at − 80 °C. Q-culture results were also correlated with VAP incidence in 13 patients when the VAP diagnosis according to the classical definition had been made on the day±1 of sample collection [3, 4].

### Enrichment-based cultures

Additional enrichment was performed by overnight incubation of a small volume (~ 100 μl) of the liquefied sample in brain heart infusion (BHI) broth followed by plating on COLOREX*™* Staph aureus as well as on blood agar plates. Presumptive *S. aureus* colonies were confirmed by MALDI-TOF (Bruker, USA).

### *nuc* gene-based qRT-PCR

200 μL of the liquefied sample was subjected to proteinase K treatment for 15 min at 56 °C followed by automated DNA extraction (NucliSENS® EasyMag ®, bioMérieux SA, France) and frozen until batched analysis by the *nuc* gene-based qPCR assay was performed. Concentrations of *S. aureus* DNA were determined using quantitative TaqMan real-time PCR targeting the single-copy *nuc* gene. qPCR was performed in a 20 μL reaction volume containing 2x Taqman™ Universal PCR Master Mix (Applied Biosystems™, California, USA), 200 nM concentrations of primers SA*nuc*F2 (TAAAGCGATTGATGGTGATACG), SA*nuc*R2 (TTCTTTGACCTTTGTCAAACTCG), TaqMan probe (cy5-TGGTCCTGAAGCAAGTGCATTTACg-BBQ) and 4 μL of DNA template. Amplification was carried out on CFX96 Touch™ Real-Time PCR detection system (Bio-Rad Laboratories Inc., California, USA) using the following cycling parameters: 5 min at 95 °C and 40 cycles of 10 s at 95 °C and 50 s at 60 °C. Bacterial loads were calculated based on a standard curve that was set up using Avogadro’s constant and the molecular weight of serially diluted *nuc* PCR product of SA NCTC 8325 [10]. Samples and standard curves were run in triplicate and samples giving Ct values ≤37 were considered positive for *S. aureus*.

## Results

### GeneXpert MRSA/SA ETA assay is a rapid and accurate method for detection of *S. aureus* in ETA samples

Additional file 1: Table S1 gives an overview of the analysed results of the 79 ETA samples utilizing SQ-culture, GeneXpert assay, Q-culture, enrichment-culture and the *nuc* gene-based qPCR. All samples positive for SQ-culture (39/79) were also positive for each of the other four methods, resulting in 100 sensitivities with SQ-culture as gold standard/comparator. The specificities of the tests, however, differed. The GeneXpert assay directly detects *S aureus* in unprocessed ETA samples based on detection of the *spa* gene by real-time amplification where a Ct value between 3 and 36 is deemed positive. With 41/79 samples positive for *S. aureus*, GeneXpert showed a 97.5% concordance with SQ-culture methods, discording for only two samples (samples 39 and 40) that were positive by GeneXpert (both with Ct values of 29) but negative by SQ-culture at 24 h (both samples grew *S. aureus* at 48 h). However, these two samples were also positive by Q-culture, enrichment-based culture and by *nuc* qPCR and were therefore assessed as positive, and along with SQ-culture results, comprised the positive samples in the extended gold standard panel (Additional file 1: Table S1). With the extended gold standard panel as a comparator, GeneXpert assay showed 100% sensitivity and specificity (Table 1). Taken together with the short total assay time (< 70 min) for GeneXpert, this assay was assessed as the best for direct detection of *S. aureus* in ETA samples in this comparator study.

**Table 1:** Sensitivities and specificities of the five methods utilized for *S. aureus* detection in ETA samples compared to the extended gold standard

		Extended gold standard*		Sensitivity (%)	Specify (%)
		+	–	(95% C1)	(95% C1)
GeneXpert	+	41	0	100	100
	–	0	38		
Q-culture	+	41	2	100	94.74
	–	0	36		(82.25–99.36)
Enrichment	+	41	3	100	92.11
	–	0	35		(78.62–98.34)
qPCR	+	41	18	100	52.63
	–	0	20		(35.82–69.02)
SQ-culture	+	39	0	95.12	100
	–	2	38	(83.47–99.40)	

*S. aureus detected by SQ-culture plus two samples that showed *S. aureus* presence by the other four methods but not by standard culture

### Quantitative and enrichment cultures have slightly higher sensitivities than semi-quantitative culture

Next, we studied Q-cultures on the chromogenic COLOREX*™* Staph aureus medium that is also commonly utilized by clinical laboratories. The sensitivity and specificity of Q-culture, calculated against the extended gold standard panel were 100 and 95%, respectively, with 43/79 ETA being positive for *S. aureus* (Table 1). Lower specificity of Q-culture was due to the fact that *S. aureus* was detected in two additional samples (samples 37 and 38 with *S. aureus* loads of 848 and 40 CFU/ml, respectively) that were negative by both SQ-culture and GeneXpert but confirmed to be positive by enrichment-based culture and by qPCR.

Enrichment-based culture identified one additional ETA sample as positive for *S. aureus* that was only confirmed by in-house qPCR. Thus, with 44/79 samples being positive after enrichment, this method showed a 100% sensitivity and 92% specificity with the extended gold standard panel as a reference. Thus, the increased sensitivities of Q-culture and enrichment culture led to lower specificity compared to SQ-culture and to GeneXpert.

**Fig. 1:**
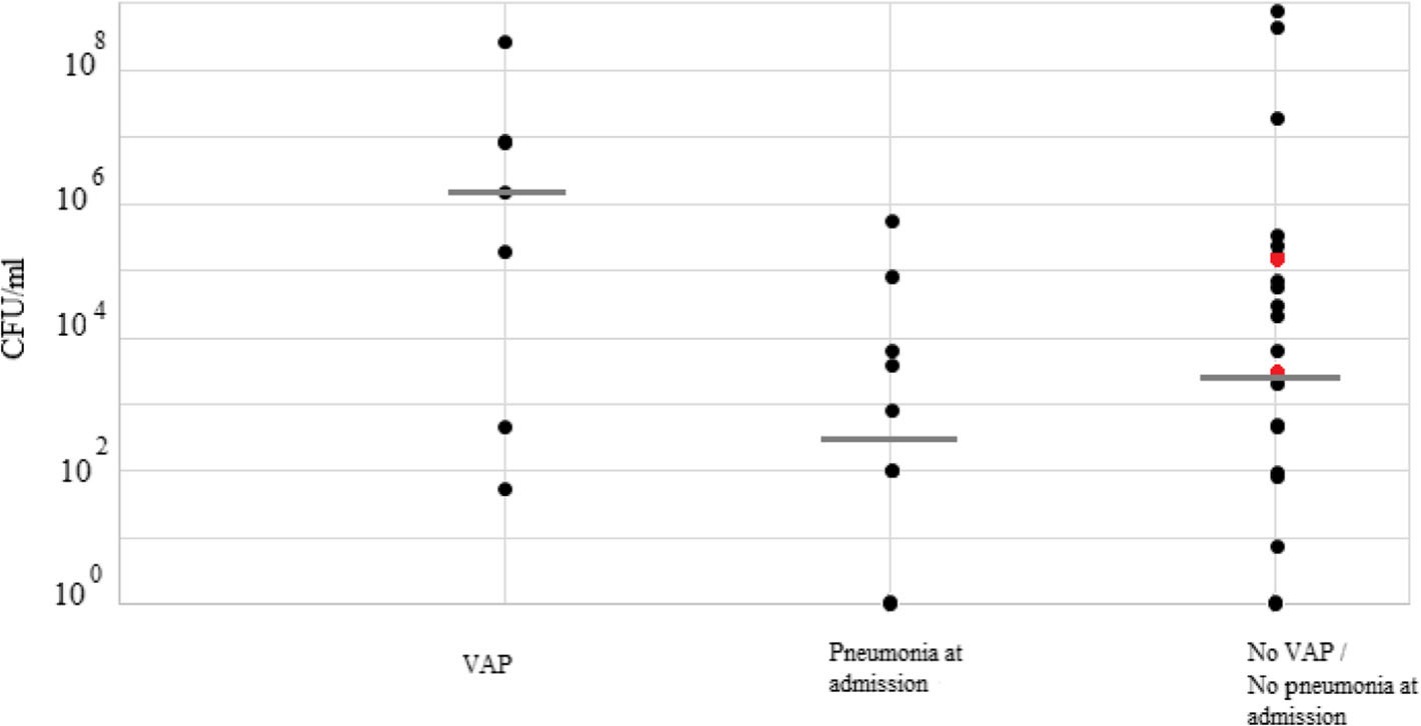
*S. aureus* loads in ETA samples from patients diagnosed with VAP on the day of sample collection or not (no VAP) or having pneumonia at ICU admission. The two red dots represent two ETAs that showed *S. aureus* presence by the other four methods but not by SQ-culture. Gray lines represent the median

Furthermore, based on the SQ-culture results, 7 patients (41; 43; 44; 50; 53; 58; 75) were diagnosed with VAP due to *S. aureus* (MSSA, *n* = 6; MRSA *n* = 1) on the day±1 of sample collection. We studied whether the *S. aureus* loads by Q-culture on COLOREX*™* Staph aureus plate were higher in samples obtained from the 7 patients who developed VAP due to *S. aureus* compared to *S. aureus*-positive patients not developing VAP. Interestingly, the *S. aureus* VAP group showed higher loads (median 1.6 × 10^6^ CFU/ml, range: 10^1^ CFU/ml − 10^8^ CFU/ml) than the *S. aureus* VAP-negative group (median 6.4 × 10^2^ CFU/ml, range: 10^0^ CFU/ml − 10^8^ CFU/ml), although the differences in loads remained non-significant (*P* = 0.806) (Fig. 1). Also, the correlation of the Ct values of the GeneXpert and qPCR was investigated. In 5 of the 7 samples, the Ct value of qPCR was higher than the Ct of the GeneXpert and one sample each, the Ct of qPCR were lower and the same as GeneXpert (*P* = 0.09) (Additional file 1: Table S1).

### In-house qPCR was the most sensitive but also the least specific assay for *S. aureus* detection in ETA samples

We also developed a *nuc* gene-based qPCR whose analytical performance was extensively validated with *S. aureus*-negative ETAs spiked with different concentrations of *S. aureus* prior to utilization in this study (data not shown). The limit of detection of the qPCR in ETAs was ~ 10^3^ CFU/ml (equals 11.4 genome equivalents detected in the total DNA input in the PCR), and the upper limit of quantification was > 10^6^ CFU/ml, and is in agreement with the diagnostic thresholds for ETA of 10^5^–10^6^ CFU/mL [11]. With this assay, 59/79 (75%) ETA samples were positive for *S. aureus* and with the extended gold standard panel as reference, showed a 100% sensitivity and 53% specificity (Table 1). Utilizing the enrichment-based culture method as a reference, that we believe was the most sensitive assay here, the sensitivity of *nuc* qPCR was 100%, however, specificity was only marginally increased to 57%. Thus, while all samples identified as *S. aureus*-positive by any of the other four methods were all detected as positive by the qPCR, an additional 15 ETA samples were also identified as positive by this method with Cts ranging from 30 to 37.

## Discussion

Early and reliable screening for *S. aureus* colonization or infection of the lower respiratory tract may inform targeted and novel preventive and therapeutic strategies. With this in mind, we studied and cross-compared 2 PCR-based and 3 culture-based methods for *S. aureus* detection in ETA samples, including a SQ-culture on blood and colistin-nalidixic-acid agar routinely used in our hospital. With the criteria of at least two methods simultaneously detecting *S. aureus*, enrichment-based culture and the in-house *nuc*-based qPCR emerged as the most sensitive methods for detection of *S. aureus* in ETA samples in this study. All 44 samples that showed the presence of *S. aureus* by enrichment-based culture were also positive by qPCR. However, of these, only 43 samples were positive by Q-culture, 41 by GeneXpert, and 39 by SQ-culture (Additional file 1: Table S1). The pre-treatment with lysomucil in the concentrations used was not inhibitory for *S. aureus* recovery here. The inhibitory role of N-acetylcysteine (NAC) to bacterial growth was shown previously [12], but we were able to show that Q-culture (NAC treated) was more sensitive than SQ- and GeneXpert (non-NAC treated). Only one sample that was *S. aureus* positive by enrichment broth was found negative by Q-culture but this sample was also negative on SQ-culture and by GeneXpert with only the q-PCR showing a positive result with a high Ct value of 38. Enrichment-based cultures also offered the best proof of *S. aureus* identification as these samples were also validated with MALDI-TOF-based *S. aureus* confirmation. The described optimized protocols for selective and enrichment-based culture methods can be employed for more sensitive *S. aureus* detection. However, the time cost in culture-based methods will always be an issue for routine clinical practice.

On the other hand, the GeneXpert assay, offering results in less than 70 min, came closest to the routine SQ-culture methods showing 97.5% agreement rate. A small discrepancy was due to 2 additional samples positive on the GeneXpert assay that were also positive by the other three methods and therefore included to comprise the extended gold standard panel. GeneXpert thus showed a 100% match with the extended gold standard panel further utilized in this study. Moreover, while SQ-ETA culture is performed on pus pockets, GeneXpert needed no sample pre-examination and, therefore, was less biased for analysis. We noted that, when taking enrichment-based culture method as reference, GeneXpert showed only 93% sensitivity. A slightly reduced sensitivity of GeneXpert might be attributable to very low doses of bacteria only detected by enrichment methods, or the presence of substances in the ETA that may interfere with PCR. The latter is supported by the fact that on two occasions (samples 45 and 68, Additional file 1: Table S1) where GeneXpert tests showed errors, a re-analysis with 2-fold sample dilution resolved the issue with high degree of positivity with Ct of 16 and 33, respectively. GeneXpert is also able to distinguish MRSA and MSSA, and the single sample that was positive for MRSA by SQ-culture results was also positive for the same on the GeneXpert assay. Previous studies showed that GeneXpert assay can be used for detection of *S. aureus* in lower respiratory tract (LRT) samples [13, 14]. In one study, 135 LRT secretions were analysed with the GeneXpert MRSA/SA skin and soft tissue infection (SSTI) assay, which showed a 99% sensitivity and 72% specificity with Q-culture as comparator [13]. Another study compared the GeneXpert MRSA nasal assay and qualitative culture for detection of MRSA in transtracheal aspirates and BAL specimens, and showed 93% concordance between the two assays [14]. The performance of the assays for MRSA detection was not assessed in the latter study [14]. The least specific assay in this study was the in-house *nuc* gene-based qPCR where 15 *S. aureus*-positive ETA samples showed no growth on any of the culture methods and were also not detected by the GeneXpert assay. The discrepancies between the quantifications from culture and the molecular methods could be attributed to different factors. For instance, while culture-negative results could arise due to prior antibiotic use or poor sample handling [15], the ability to detect bacteria at low concentrations by qPCR, even from extracellular DNA, could make this test highly non-specific. These findings are in line with previously published studies [16–18]. Interestingly, GeneXpert, also a qPCR method poses no such problem and is most likely due to its capability to detect only whole bacteria through a filter and a washing step that removes extracellular *S. aureus* DNA. Lastly, GeneXpert was the only test that was capable of drastically decreasing the critical time-to-initiation of any potential preventive or therapeutic strategy against VAP, while additionally also allowing detection of MRSA, an important criterion for selecting the right antibiotic.

## Conclusion

*S. aureus* is a common cause of VAP, a common nosocomial infection associated with a substantial increase in morbidity, mortality as well as in a costly prolongation of patient bed days. With the knowledge that prior colonization with potential pathogens, such as *S. aureus*, plays a significant role in the development of nosocomial infections, rapid and accurate detection of lower respiratory tract colonization and/or infection with *S. aureus* may inform targeted preventive and therapeutic strategies. A recently introduced molecular test, currently utilized for research purposes only, that directly detects *S. aureus* from ETA samples is the GeneXpert SA/MRSA ETA assay. Here, we compared 2 PCR-based (including GeneXpert) and three culture-based methods for *S. aureus* detection in ETAs collected from mechanically-ventilated patients. Although this is a one-centre study on a limited number of samples, we show here for the first time that GeneXpert MRSA/SA ETA is a rapid and sensitive method for *S. aureus* detection in ETA samples. In centres utilizing culture methods, we would recommend increasing assay sensitivity by introducing enrichment-based culture in addition to direct SQ-culture.

## Additional file

Additional file 1: **Table S1.** Overview of the results obtained on 79 ETAs using SQ-culture, the GeneXpert assay, Q-culture, enrichment-based culture, and in-house *nuc* gene-based qPCR. 0-4: negative (0), light (1), moderate (2) and heavy (3). (PDF 88 kb)

## Abbreviations

BHI: brain heart infusion
CFU: colony forming units
CNA: colistin-nalidixic acid agar
ETA: endotracheal aspirates
ICU: intensive care unit
LRT: lower respiratory tract
*mecA*: methicillin resistance gene
MSSA and MRSA: methicillin-sensitive and -resistant *Staphylococcus aureus*
NAC: N-acetylcysteine
nuc: thermostable nuclease
PBS: phosphate buffered saline
Q: quantitative
*S. aureus*: *Staphylococcus aureus*
SCC*mec*: staphylococcal cassette chromosome
spa: staphylococcal protein A
SQ: semi-quantitative
SSTI: skin and soft tissue infection
VAP: ventilator-associated pneumonia

## References

[1] Lee AS, de Lencastre H, Garau J, Kluytmans J, Malhotra-Kumar S, Peschel A, et al. Methicillin-resistant *Staphylococcus aureus*. Nat Rev Dis Prim [Internet]. 2018 May 31;4:18033. Available from: http://www.nature.com/articles/nrdp201833 10.1038/nrdp.2018.33 29849094

[2] Chastre J, Fagon J-Y (2002). Ventilator-associated pneumonia. Am J Respir Crit Care Med.

[3] American Thoracic Society ; Infectious Diseases Society of America (2005). Guidelines for the management of adults with hospital-acquired, ventilator-associated, and healthcare-associated pneumonia. Am J Respir Crit Care Med.

[4] Jorens PG (2016). Sticking to an old definition of ventilator-associated pneumonia is not old-fashioned. Respir Care [Internet].

[5] Bonten MJ, Weinstein RA. The role of colonization in the pathogenesis of nosocomial infections. Infect Control Hosp Epidemiol [Internet]. 1996;17(3):193–200. Available from: http://www.ncbi.nlm.nih.gov/pubmed/8708364 10.1086/647274 8708364

[6] Paling FP, Wolkewitz M, Bode LGM, Klein Klouwenberg PMC, Ong DSY, Depuydt P, et al. *Staphylococcus aureus* colonization at ICU admission as a risk factor for developing S.?aureus ICU pneumonia. Clin Microbiol Infect [Internet]. 2017 Jan;23(1):49.e9–49.e14. Available from: http://linkinghub.elsevier.com/retrieve/pii/S1198743X16304281 10.1016/j.cmi.2016.09.022 27693658

[7] Brusselaers N, Labeau S, Vogelaers D, Blot S. Value of lower respiratory tract surveillance cultures to predict bacterial pathogens in ventilator-associated pneumonia: systematic review and diagnostic test accuracy meta-analysis. Intensive Care Med [Internet]. 2013;39(3):365–375. Available from: http://www.ncbi.nlm.nih.gov/pubmed/23188467 10.1007/s00134-012-2759-x 23188467

[8] Garcia LS, Isenberg HD (2010). Clinical microbiology procedures handbook.

[9] Wolk DM, Struelens MJ, Pancholi P, Davis T, Della-Latta P, Fuller D, et al. Rapid Detection of *Staphylococcus aureus* and Methicillin-Resistant *S. aureus* (MRSA) in Wound Specimens and Blood Cultures: Multicenter Preclinical Evaluation of the Cepheid Xpert MRSA/SA Skin and Soft Tissue and Blood Culture Assays. J Clin Microbiol [Internet]. 2009 Mar 1;47(3):823–826. Available from: http://jcm.asm.org/cgi/doi/10.1128/JCM.01884-08 10.1128/JCM.01884-08 PMC2650929 19144803

[10] Fey A, Eichler S, Flavier S, Christen R, Höfle MG, Guzmán CA. Establishment of a real-time PCR-based approach for accurate quantification of bacterial RNA targets in water, using Salmonella as a model organism Appl Environ Microbiol [Internet]. 2004 Jun;70(6):3618–3623. Available from: http://www.ncbi.nlm.nih.gov/pubmed/15184165 10.1128/AEM.70.6.3618-3623.2004 PMC427797 15184165

[11] Torres A (2006). The new American Thoracic Society/infectious disease Society of North America guidelines for the management of hospital-acquired, ventilator-associated and healthcare-associated pneumonia: a current view and new complementary information. Curr Opin Crit Care [Internet].

[12] Eroshenko D, Polyudova T, Korobov V (2017). N-acetylcysteine inhibits growth, adhesion and biofilm formation of gram-positive skin pathogens. Microb Pathog [Internet].

[13] Cercenado E, Marin M, Burillo A, Martin-Rabadan P, Rivera M, Bouza E (2012). Rapid detection of Staphylococcus aureus in lower respiratory tract secretions from patients with suspected ventilator-associated pneumonia: evaluation of the Cepheid Xpert MRSA/SA SSTI assay. J Clin Microbiol [Internet].

[14] Oh A-c, Lee jk, Lee Hn, Hong Yj, Chang Yh, Hong S-i, et al. Clinical utility of the Xpert MRSA assay for early detection of methicillin-resistant *Staphylococcus aureus*. Mol Med Rep [Internet]. 2013 Jan;7(1):11–15. Available from: https://www.spandidos-publications.com/10.3892/mmr.2012.1121 10.3892/mmr.2012.1121 PMC3572728 23064681

[15] Wolk DM, Picton E, Johnson D, Davis T, Pancholi P, Ginocchio CC, et al. Multicenter Evaluation of the Cepheid Xpert Methicillin-Resistant *Staphylococcus aureus* (MRSA) Test as a Rapid Screening Method for Detection of MRSA in Nares. J Clin Microbiol [Internet]. 2009 Mar 1;47(3):758–764. Available from: http://jcm.asm.org/cgi/doi/10.1128/JCM.01714-08 10.1128/JCM.01714-08 PMC2650940 19129414

[16] Lacroix M, Barraud O, Clavel M, Filiputti D, Prudent S, François B, et al. Rapid quantification of *Staphylococcus aureus* from endotracheal aspirates of ventilated patients: a proof-of-concept study. Diagn Microbiol Infect Dis [Internet]. 2015 Oct;83(2):117–120. Available from: http://linkinghub.elsevier.com/retrieve/pii/S0732889315002175 10.1016/j.diagmicrobio.2015.06.014 26227327

[17] Rios-Licea MM, Bosques FJ, Arroliga AC, Galindo-Galindo JO, Garza-Gonzalez E (2010). Quadruplex real-time quantitative PCR assay for the detection of pathogens related to late-onset ventilator-associated pneumonia. J Microbiol Methods [Internet].

[18] Alonzo TA, Pepe MS. Using a combination of reference tests to assess the accuracy of a new diagnostic test. Stat Med [Internet]. 1999 Nov 30;18(22):2987–3003. Available from: http://www.ncbi.nlm.nih.gov/pubmed/10544302 10.1002/(sici)1097-0258(19991130)18:22<2987::aid-sim205>3.0.co;2-b 10544302

