# Supplementary material for: Comparison of GeneXpert MRSA/SA ETA assay with semi-quantitative and quantitative cultures and nuc gene-based qPCR for detection of Staphylococcus aureus in endotracheal aspirate samples
Source: Antimicrob Resist Infect Control. 2019 Jan 5;8:4. doi: 10.1186/s13756-018-0460-8 (PMC6321727; doi:10.1186/s13756-018-0460-8)
Supplement: Supplementary file 1 — Table S1. Overview of the results obtained on 79 ETAs using SQ-culture, the GeneXpert assay, Q-culture, enrichment-based culture, and in-house nuc gene-based qPCR. 0-4: negative (0), light (1), moderate (2) and heavy (3). (PDF 88 kb) [file 13756_2018_460_MOESM1_ESM.pdf]

**Supplementary Table 1**: Overview of the results obtained on 79 ETAs using SQ-culture, the GeneXpert assay, Q-culture, enrichment-based culture, and in-house *nuc* gene-based qPCR. 0-4: negative (0), light (1), moderate (2) and heavy (3).

| Sample ID | SQ-culture method | | Extended gold standard# | GeneXpert assay | | Q-culture | | Enrichment-based culture | *Nuc* gene-based qPCR | |
|---|---|---|---|---|---|---|---|---|---|---|
| | *S. aureus* detected* | 0-4 | | *S. aureus* detected | Ct value | *S. aureus* detected | CFU/ml | *S. aureus* detected | *S. aureus* detected | Ct value |
| **1** | - | 0 | - | - | 39 | - | 0,00E+00 | - | - | 38 |
| **2** | - | 0 | - | - | 39 | - | 0,00E+00 | - | - | 38 |
| **3** | - | 0 | - | - | 39 | - | 0,00E+00 | - | - | 38 |
| **4** | - | 0 | - | - | 39 | - | 0,00E+00 | - | - | 38 |
| **5** | - | 0 | - | - | 39 | - | 0,00E+00 | - | - | 38 |
| **6** | - | 0 | - | - | 39 | - | 0,00E+00 | - | - | 38 |
| **7** | - | 0 | - | - | 39 | - | 0,00E+00 | - | - | 38 |
| **8** | - | 0 | - | - | 39 | - | 0,00E+00 | - | - | 38 |
| **9** | - | 0 | - | - | 39 | - | 0,00E+00 | - | - | >38 |
| **10** | - | 0 | - | - | 39 | - | 0,00E+00 | - | - | >38 |
| **11** | - | 0 | - | - | 39 | - | 0,00E+00 | - | - | >38 |
| **12** | - | 0 | - | - | 39 | - | 0,00E+00 | - | - | >38 |
| **13** | - | 0 | - | - | 39 | - | 0,00E+00 | - | - | >38 |
| **14** | - | 0 | - | - | 39 | - | 0,00E+00 | - | - | >38 |
| **15** | - | 0 | - | - | 39 | - | 0,00E+00 | - | - | >38 |
| **16** | - | 0 | - | - | 39 | - | 0,00E+00 | - | - | >38 |
| **17** | - | 0 | - | - | 39 | - | 0,00E+00 | - | - | >38 |
| **18** | - | 0 | - | - | 36 | - | 0,00E+00 | - | - | >38 |
| **19** | - | 0 | - | - | 39 | - | 0,00E+00 | - | - | >38 |
| **20** | - | 0 | - | - | 38 | - | 0,00E+00 | - | - | 38 |
| **21** | - | 0 | - | - | 39 | - | 0,00E+00 | - | + | 25 |
| **22** | - | 0 | - | - | 39 | - | 0,00E+00 | - | + | 30 |
| **23** | - | 0 | - | - | 39 | - | 0,00E+00 | - | + | 31 |
| **24** | - | 0 | - | - | 39 | - | 0,00E+00 | - | + | 34 |
| **25** | - | 0 | - | - | 39 | - | 0,00E+00 | - | + | 35 |
| **26** | - | 0 | - | - | 39 | - | 0,00E+00 | - | + | 35 |
| **27** | - | 0 | - | - | 39 | - | 0,00E+00 | - | + | 35 |
| **28** | - | 0 | - | - | 39 | - | 0,00E+00 | - | + | 35 |
| **29** | - | 0 | - | - | 39 | - | 0,00E+00 | - | + | 36 |
| **30** | - | 0 | - | - | 39 | - | 0,00E+00 | - | + | 36 |
| **31** | - | 0 | - | - | 39 | - | 0,00E+00 | - | + | 36 |
| **32** | - | 0 | - | - | 39 | - | 0,00E+00 | - | + | 36 |
| **33** | - | 0 | - | - | 39 | - | 0,00E+00 | - | + | 37 |
| **34** | - | 0 | - | - | 39 | - | 0,00E+00 | - | + | 37 |
| **35** | - | 0 | - | - | 39 | - | 0,00E+00 | - | + | 37 |
| **36** | - | 0 | - | - | 39 | - | 0,00E+00 | + | + | >38 |
| **37** | - | 0 | - | - | 39 | + | 8,48E+02 | + | + | 29 |
| **38** | - | 0 | - | - | 39 | + | 4,00E+01 | + | + | 35 |
| **39** | - | 0 | + | + | 29 | + | 3,00E+03 | + | + | 30 |
| **40** | - | 0 | + | + | 29 | + | 1,77E+05 | + | + | 32 |
| **41** | + | 1 | + | + | 31 | + | 5,60E+01 | + | + | 24 |
| **42** | + | 1 | + | + | 34 | + | 8,64E+02 | + | + | 27 |
| **43** | + | 3 | + | + | 19 | + | 8,56E+06 | + | + | 19 |
| **44** | + | 3 | + | + | 17 | + | 2,65E+08 | + | + | 21 |
| **45** | + | 3 | + | + | 16 | + | 7,80E+08 | + | + | 21 |
| **46** | + | 2 | + | + | 18 | + | 2,04E+07 | + | + | 24 |
| **47** | + | 3 | + | + | 14 | + | 4,39E+08 | + | + | 24 |
| **48** | + | 1 | + | + | 24 | + | 6,05E+04 | + | + | 25 |
| **49** | + | 3 | + | + | 22 | + | 1,59E+05 | + | + | 25 |
| **50** | + | 3 | + | + | 19 | + | 1,56E+06 | + | + | 25 |
| **51** | + | 1 | + | + | 21 | + | 5,12E+02 | + | + | 25 |
| **52** | + | 2 | + | + | 26 | + | 9,44E+02 | + | + | 26 |
| **53** | + | 2 | + | + | 23 | + | 1,92E+05 | + | + | 26 |
| **54** | + | 2 | + | + | 26 | + | 6,42E+03 | + | + | 27 |
| **55** | + | 1 | + | + | 25 | + | 7,38E+04 | + | + | 27 |
| **56** | + | 1 | + | + | 28 | + | 3,51E+05 | + | + | 27 |
| **57** | + | 1 | + | + | 24 | + | 2,40E+05 | + | + | 27 |
| **58** | + | 3 | + | + | 22 | + | 9,31E+06 | + | + | 27 |
| **59** | + | 1 | + | + | 30 | + | 9,60E+01 | + | + | 27 |
| **60** | + | 2 | + | + | 31 | + | 0,00E+00 | + | + | 28 |
| **61** | + | 2 | + | + | 25 | + | 5,71E+05 | + | + | 28 |
| **62** | + | 1 | + | + | 34 | + | 8,80E+01 | + | + | 28 |
| **63** | + | 2 | + | + | 26 | + | 0,00E+00 | + | + | 28 |
| **64** | + | 1 | + | + | 27 | + | 1,04E+02 | + | + | 29 |
| **65** | + | 2 | + | + | 25 | + | 3,16E+04 | + | + | 29 |
| **66** | + | 3 | + | + | 24 | + | 1,47E+05 | + | + | 30 |
| **67** | + | 3 | + | + | 30 | + | 2,59E+03 | + | + | 31 |
| **68** | + | 2 | + | + | 33 | + | 0,00E+00 | + | + | 31 |
| **69** | + | 1 | + | + | 30 | + | 8,00E+00 | + | + | 32 |
| **70** | + | 1 | + | + | 28 | + | 1,04E+02 | + | + | 32 |
| **71** | + | 1 | + | + | 30 | + | 2,06E+03 | + | + | 32 |
| **72** | + | 1 | + | + | 35 | + | 0,00E+00 | + | + | 33 |
| **73** | + | 1 | + | + | 31 | + | 3,99E+03 | + | + | 33 |
| **74** | + | 3 | + | + | 27 | + | 6,69E+03 | + | + | 35 |
| **75** | + | 1 | + | + | 30 | + | 4,88E+02 | + | + | 36 |
| **76** | + | 1 | + | + | 29 | + | 2,14E+04 | + | + | 36 |
| **77** | + | 1 | + | + | 32 | + | 4,80E+02 | + | + | 36 |
| **78** | + | 1 | + | + | 34 | + | 0,00E+00 | + | + | 36 |
| **79** | + | 2 | + | + | 27 | + | 8,32E+04 | + | + | 31 |

* +/-: Sample detected positive or negative for *S. aureus*

\# *S. aureus* detected by standard culture plus two samples that showed *S. aureus* presence by the other four methods but not by SQ-culture

0-4: Growth in four quadrants of SQ-culture. CFU: colony forming units
